# Intraocular neutralizing antibodies against aflibercept in patients with age related macular degeneration

**DOI:** 10.1186/s12886-022-02761-9

**Published:** 2023-01-10

**Authors:** Andrea Gyenes, Lilla István, Fruzsina Benyó, András Papp, Miklós Resch, Noémi Sándor, Mihály Józsi, Zoltán Z. Nagy, Illés Kovács, Szilárd Kiss

**Affiliations:** 1 Department of Ophthalmology, Semmelweis University, Budapest, Hungary; 2 Department of Immunology, ELTE Eötvös Loránd University, Budapest, Hungary; 3 MTA-ELTE Complement Research Group, Eötvös Loránd Research Network (ELKH), Budapest, Hungary; 4 Department of Ophthalmology, Weill Cornell Medical College, New York, USA; 5 Department of Clinical Ophthalmology, Faculty of Health Sciences, Semmelweis University, Budapest, Hungary

**Keywords:** Age related macular degeneration, anti-VEGF, Treatment

## Abstract

**Purpose:**

To detect immunoglobulins in aqueous humour of AMD patients after repeated administration of intravitreal aflibercept.

**Patients and methods:**

Twenty-one patients (age: 77.85 ± 9.21 years) previously treated with intravitreal aflibercept due to wet type age-related macular degeneration (AMD group) and 18 age-matched control subjects (age: 69.75 ± 12.67 years) were included in this study. Patients in the AMD group received a mean of 5 intravitreal injections (min: 1 max: 17) prior to the cataract surgery. Samples of aqueous humour (50 μl) were obtained by anterior chamber paracentesis as the first step of routine cataract surgery. The IgG content of the samples was analysed by an in-house developed ELISA system.

**Results:**

A significant increase in nonspecific IgG levels in the AMD group was detected compared to the control group (13.37 ± 6.65 vs. 9.44 ± 6.55 μg/ml; *p* = 0.03). In 11 patients, intraocular anti-aflibercept immunoglobulins could be detected (0.05 ± 0.01 μg/ml) which was significantly higher than the limit of detection for anti-aflibercept (0.04 μg/ml; *p* = 0.001). No correlation was found between the number of injections or the type of CNV and the aqueous level of anti-aflibercept (*r* = 0.02; *p* = 0.95).

**Conclusion:**

According to our results, penetration of non-specific systemic antibodies through the impaired blood-retinal barrier is higher in patients with neovascular AMD than in subjects with an intact structural barrier. Evaluation of neutralizing antibodies to anti-VEGF agents in the aqueous humour can lead us to understanding tachyphylaxis and changes in intraocular immune mechanisms due to AMD.

## Introduction

Age related macular degeneration (AMD) is the leading cause of visual impairment [1, 2]. Approximately 30–50 million people are affected by AMD worldwide [3]. Visual impairment due to AMD has a significant effect on health related quality of life [4]. It is associated with increased morbidity, partly from higher risk of falls [5, 6]. AMD affects quality of life not only related to tasks requiring good vision such as reading, but also people’s ability to participate in social interactions, leisure and other chosen activities [7]. Social issue of AMD encompasses the influence on emotional health, psychological distress, associated depression, limited interactions and social dependence [8–11]. The two major forms of the disease are neovascular and dry AMD. VEGF is a key player in neovascular AMD [12]. Repeated administration of anti-VEGF agents are standard treatment for AMD. Aflibercept (Eylea, Regeneron Pharmaceuticals, Inc., Tarrytown, NY) is a recombinant fusion protein that binds to all VEGF-A, VEGF-B isoforms and growth factor [13, 14], and is the third approved anti-VEGF agent. Its binding affinity for VEGF-A is higher than that of bevacizumab or ranimizumab. Although the majority of patients respond well to the anti-VEGF therapy, there are patients, who do not show any anatomical or functional improvement from anti-VEGF therapy. These non–responders constitute 14–25% of AMD patients [15–17]. The response to anti-VEGF therapy has been found to be influenced by the patient’s age, disease duration, baseline BCVA, the presence of particular genotype risk alleles and also some anatomical findings [18]. Zuber-Laskawiec et al. reported about 22.2% of total patients to be non-responders to anti-VEGF and the main anatomical predictor was presence of serous PED in lack of positive reaction [19].

Tachyphylaxis is a not fully understood therapeutic problem, which can be defined as a poor, weakening response, after an initial positive reaction to the treatment. Tachyphylaxis to anti-VEGF therapy has been reported to occur at a rate of 10% for bevacizumab [20] and a rate of 7.7% for ranimizumab [21, 22]. It is known, that some eyes with AMD develop tachyphylaxis on intravitreal aflibercept as well [23]. Tachyphylaxis on intravitreal aflibercept was reported in 8.9% of all patients with treatment-naive exsudative AMD during the study period and at annual rate of 2.2–4.0% [24]. This corresponds with the results of Eghoj, who reported about tachyphylaxis in 2% of treated eyes during the first year of treatment [25].

According to previous reports and clinical experiences switching from one type of intravitreal drug to another may be effective for eyes that develop tachyphylaxis. Switching to another anti-VEGF agent is helpful in restoring the efficacy of a drug, as it can serve as a drug holiday in patients with tachyphylaxis [26]. Gasperini et al. reported successful switch between ranimizumab and bevacizumab or vice versa when tachyphylaxis developed on one drug [27]. It has been shown, that aflibercept is a successful treatment option for patients, who are not responsive or developed tachyphylaxis on ranimizumab or bevacizumab. Despite the fact, that ranimizumab has weaker pharmacological effect than aflibercept, which has higher affinity for VEGF-A and it occupies VEGF-B and placental growth factor receptors, switch from aflibercept to ranimizumab can be effective in some cases [28–30]. In cases of tachyphylaxis on aflibercept, exsudative changes improved in 5 of 9 eyes when switching aflibercept to ranimizumab [31]. A possible explanation for this phenomenon can be the presence of intraocular anti-aflibercept antibodies [32] due to repeated intravitreal injections. Resistence to a drug from immunization has already been described, and is well known for infliximab and adalimumab monoclonal antibody therapies in rheumatoid arthritis and Crohn’s disease [33]. The presence of neutralizing antibodies in the blood due to immunization to ranibizumab has already been reported [34], in fact, systemic antibodies were detected in 17% of patients treated with intravitreal ranimizumab [35]. However, a subgroup analysis of MARINA study investigating the clinical difference between patients with and without systemic antibodies to ranimizumab did not find a plausible relationship between the presence of systemic antibodies and response to anti-VEGF therapy [36].

Although the blood retina barrier normally serves as a physical barrier, which maintains ocular immune privilege [37], it is documented that in AMD these barriers are damaged and immune suppressive pathways are altered or lost [38]. The purpose of the present study was to examine aqueous samples of AMD patients treated with intravitreal aflibercept for the presence of nonspecific as well as specific anti-aflibercept immunoglobulins.

## Patients and methods

### Patients

Twenty one patients (age: 77.85 ± 9.21 years; male: 12, female: 9) previously treated with intravitreal aflibercept due to wet AMD (AMD group) scheduled for cataract surgery, and 16 age-matched control subjects (age: 69.75 ± 12.67 years; male: 9, female: 9) with cataract but without any history of retinal or uveal diseases (control group) were included in this study. Patients in the AMD group received a mean of 5 intravitreal injections (min: 1 max: 17) prior to the cataract surgery. The baseline characteristics of patients are summarized in Table 1 (Baseline OCT, last OCT, type of wet AMD, etc).

**Table 1:** Non-specific and anti-aflibercept immunglobulin levels in the two study groups

Immunglobulins in aqueous samples	Control group	AMD group	p
Non-specific IgG (μg/ml)	9.44 ± 6.55	13.37 ± 6.65	0.03
Anti-aflibercept IgG (μg/ml)	0.02 ± 0.01	0.05 ± 0.01	< 0.001

### Aqueous sample collection

Samples of aqueous humour (50 μl) were obtained by anterior chamber paracentesis as the first step of routine cataract surgery under surgical microscope. Restoration of anterior chamber depth was performed by anterior chamber infusion of balanced salt solution. Samples were transmitted into an Eppendorf tube and were stored frozen at − 20 °C until cytokine profiling.

### Immune ELISA

The IgG content of the samples was analysed by an in-house developed ELISA system. To determine whether the samples contain any IgG, high-binding 96 well ELISA plates (Sarstedt) were coated with anti-human IgG Fc antibody (Sigma) overnight at 4 °C. After that wells were blocked with PBS-1%BSA (PanReac). Samples were diluted 50x in PBS-1%BSA and added to the wells for 1 h at RT. Bound IgG or anti-aflibercept-IgG complexes were detected by HRP-conjugated anti-human IgG Fab (Sigma). In this setup both the „naked” IgG and – if present- the Eylea-IgG complexes are detected. However, no signal is observed if no IgG is present in the sample. For positive control, purified human IgG1 (Sigma) was used and buffer control served as negative control.

To determine if the IgG found in the patients sample is specific to Eylea, 96 well high-binding ELISA plates were coated with 10 μg/ml aflibercept overnight at 4 °C. After blocking with PBS-1%BSA the samples were added for 1 h at RT. IgG bound to aflibercept were detected with HRP-conjugated anti-human IgG Fab (Sigma) which does not recognize aflibercept. Coating efficiency was verified by incubating the aflibercept coat with HRP-conjugated anti-human IgG Fc. Both ELISA were developed with TMB (BioLegend) and reaction was stopped by adding H_2_SO_4_ (Sigma) to the wells. Absorbance values were determined with Thermo Multiskan EX spectrophotometer at 450 nm and 620 nm as control.

### Statistical analysis

Statistical analysis was performed by using the Statistica software (version 13.0, TIBCO, Palo Alto, CA, USA). The Shapiro-Wilks W-test was used to test the normality of the data. The Mann-Whitney U test and the Student’s t-test on independent samples were used to analyse the differences between the results obtained from the two study groups. In all statistical analyses, a *P*-value of less than 0.05 was considered to be statistically significant.

## Results

**Fig. 1:**
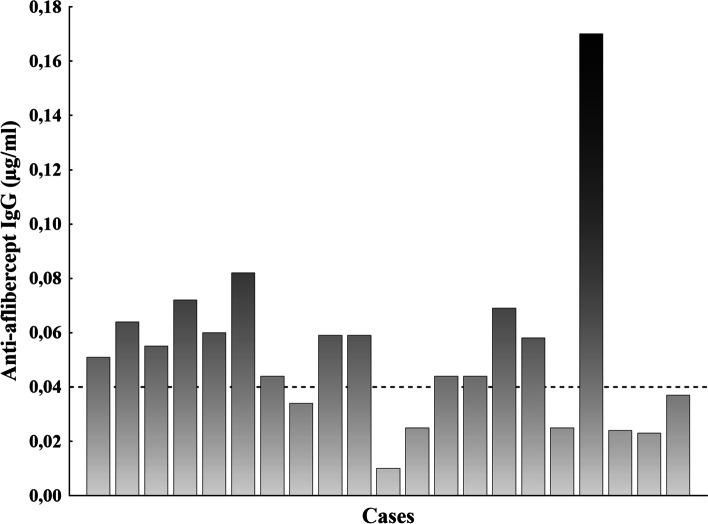
Anti-aflibercept values in the samples and the limit of detection (dotted line)

**Fig. 2:**
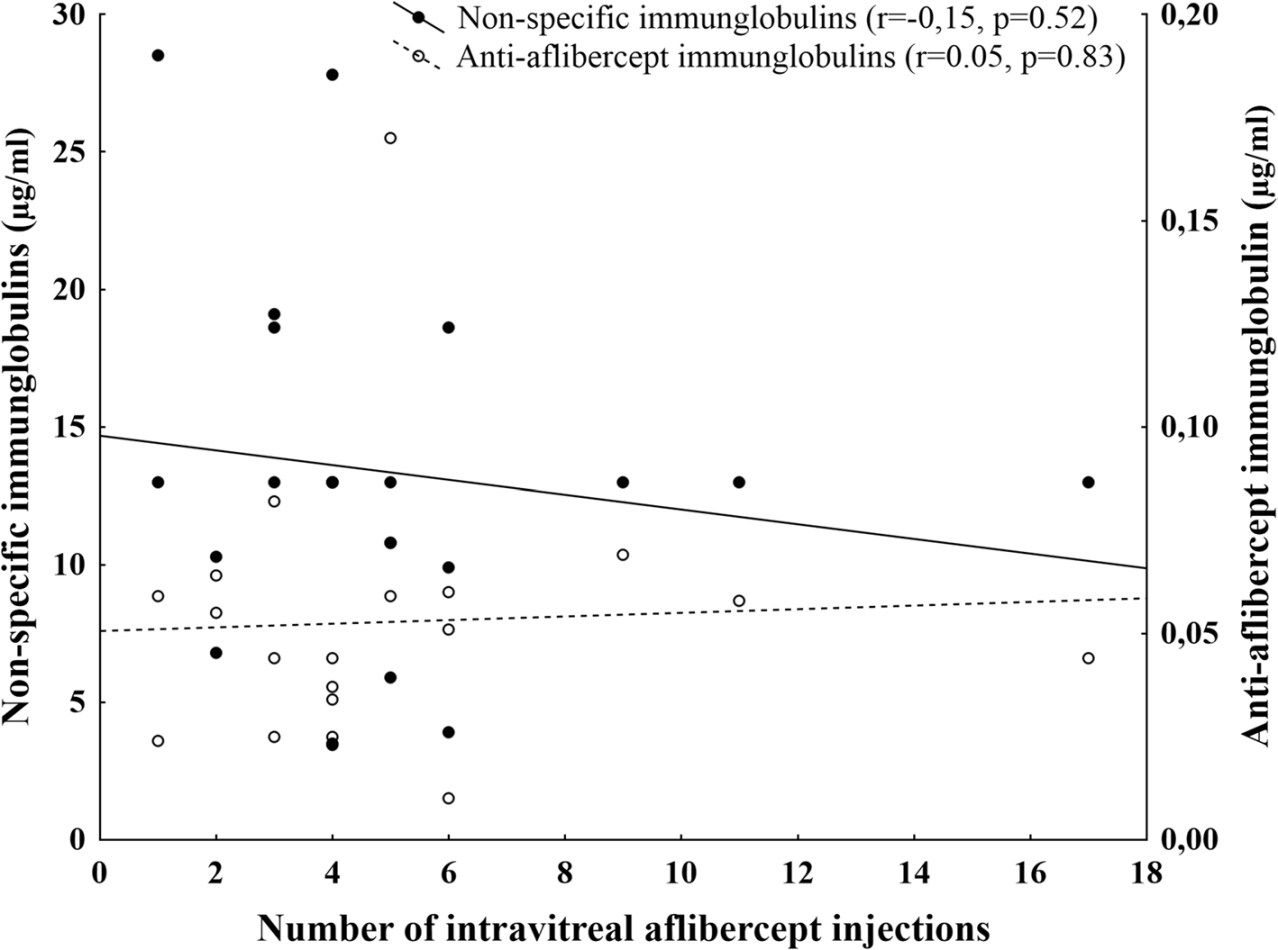
Correlation of the number of intravitreal injections with the aqueous level non-specific and specific anti-aflibercept immunglobulins

Regarding the effect of intravitreal aflibercept treatment; 5 patient from the AMD group developed weakening response (tachyphylaxis) after repeated aflibercept therapy. We detected both non-specific IgG as well as anti-aflibercept IgGs in control samples, however there was a significantly increased level of both nonspecific and specific antibody levels in samples of the AMD group compared to the control group (Table 1). While non-specific IgGs are considered to be a normal finding in aqueous samples of normal subjects, the small level of anti-aflibercept in control subjects can be considered as non-specific background of the ELISA measurement, and does not indicate the presence of specific immunglobulins in these samples. After setting the limit of detection for anti-aflibercept at 0.04 μg/ml (mean + 2SD from control samples) there were 11 patients in the AMD group, whose anti-aflibercept level exceeded this threshold and can be considered as true positive results (Fig. 1). In these 11 patients, the level of specific anti-aflibercept IgGs showed no correlation with the number of injections (*r* = 0.02; *p* = 0.95) or the development of tachyphilaxis. Similarly, we found no correlation between the number of injections and the aqueous level of non specific or specific immunoglubulin levels when analysing the whole cohort (Fig. 2).

## Discussion

In this study, significantly elevated level of non-specific antibodies were detected in aqueous samples of wet-AMD patients previously treated with aflibercept compared to subjects with no history of retinal or uveal diseases. These results suggest, that the penetration of non-specific systemic antibodies through the impaired blood-retinal barrier is higher in patients with neovascular AMD than in subjects with an intact structural barrier. These results can be informative in understanding changes in intraocular immune mechanisms due to AMD in which blood-retinal barrier permeability is increased and both intravitreally administered drugs and systemic auto-antibodies can cross this barrier with ease. The presence of specific anti-aflibercept antibodies in a subset of AMD patients, suggests that at least in some cases systemic exposure to intravitreal aflibercept results in the production of anti-aflibercept antibodies which can enter the eye through the compromised blood retinal barrier.

Aging is an important risk factor in the development of degenerative and angiogenic diseases, such as AMD and immune protection that maintains retinal immune privilege also undergoes changes with the aging process. Aging influences the mobility of microglia, which reduces its phagocytic function and immune regulation is shifted toward a proinflammatory state. Expression of complement inhibitors is reduced, while the level of complement activators is increased in the aging retina. Physical barriers like inner and outer BRB appeared to be weakened due to age–related changes. Moreover, it has been described, that AMD promotes the breakdown of blood retina barrier. Dysregulated parainflammation in AMD weakens the blood retina barrier, resulting in the damage of retinal-immune privilege [39, 40]. It has already been speculated, that altered immune privilege in AMD may negatively affect the potential of drugs or genes that are administered intravitreally or subretinally [41].

The eye is an immune-privileged organ, meaning an extreme form of regional immunity [42]. The nonfenestrated capillaries of retinal vessels and the retinal pigment epithelium serve a membrane function with tight junctions [43]. Blood retinal barrier serves the integrity of immune privilege, as a barrier it limits the ingress of blood-borne molecules and cells [44–46].

Anterior chamber associated immune deviation (ACAID) belongs to mechasmisms of immune privilege as well [47–54]. Evaluating the integrity of BRB is necessary to monitor the efficacy of different therapeutic strategies [55]. Opening of tight junctions, upregulation of vesicular transport, degenerative changes of RPE, RVE, pericytes and perivascular astrocytes by several mediators are shown to be the major contributors to BRB failure.

The factors which can influence the efficacy of anti-VEGF drugs in neovascular AMD are not completely clear, and several possible explanations were reported such as increased expression of VEGF and its receptors [56], changes in signal transduction that can stimulate the expansion of CNV [57] and development of a systemic immune response that can lead to tachyphylaxis [58].

Previously Hara et al. described a connection between the localization of CNV and development of tachyphylaxis, as in their cohort all eyes with tachyphylaxis had CNV beneath the RPE and were significantly less likely to show intraretinal edema on OCT [59].

Evaluation of neutralizing antibodies to anti-VEGF agents in the aqueous humour can lead us to understanding tachyphylaxis and could serve as a potential biomarker for the response to anti-VEGF treatment in addition to the current practice of studying retinal morphology. The presence of aflibercept-specific neutralizing antibodies in the aqueous humour could indicate a defect in intraocular immune privilege and initiation of cell mediated immune response. These neutralizing antibodies can inhibit anti-VEGF molecules from consequent intravitreal injections to reach their target, and thus, suppressing the effectivity of long term treatment.

According to our observations, the level of intravitreal antibodies is not correlated to the number of injections, which corresponds to the results from Hara [60]. It has been shown, that there is no time period during which tachyphylaxis is more likely to occur and the risk of tachyphylaxis does not increase during long-term treatment [61], in contrary to drug tolerance which is defined as a significant decrease in response in a long term administration [62]. Eyes that had been treated with intravitreal aflibercept 6–10 times or more than 16 times developed tachyphylaxis about 6%, whereas those treated 11–15 times were less likely to develop tachyphylaxis (1.6 and 3.4%) respectively [24]. It is also known, that tachyphylaxis cannot be overcome by increasing the dose of the drug [63]. It should be mentioned, that treatment efficacy to anti-VEGF in AMD patients is also determined by genetic factors which can modulate the drug response [64].

Interesting consideration is how neutralizing antibodies might affect intravitreal gene delivery to treat AMD. Pre-existing immunity can inhibit the transduction of Adeno-Associated Virus (AAV) vectors in immune privileged regions, such as the eye [65]. Humoral immunological memory can inhibit AAV gene delivery by host antibodies [66] and T cell mediated immune response to vector proteins can block the transduced cells [67]. The presence of baseline neutralizing antibodies limits the effectiveness of gene therapy using intravitreal adenovirus vectors [68–71]. Both intravitreally delivered gene therapy vectors and the produced anti-VEGF agent can be susceptible to neutralizing antibodies present in the ocular fluids [72, 73].

As a limitation of our study, there is a lack of additional control group; patients with wet AMD without intravitreal injections. These patients could theoretically serve as a control group as they have compromised blood retinal barrier and elevated level of non-specific antibodies in aqueous humour, but in reality, there were no patients enrolled for a cataract surgery with untreated wet AMD. This is due to the fact that every patient enrolled for a cataract surgery undergo funduscopy as well; if signs of wet AMD can be seen, the treatment protocol is initiated with intravitreal injections according to standard of care. The only exception to this rule is patients with mature cataract who cannot be examined with funduscopy and OCT. As a further limitation of our study, our data is obtained from a cohort taken from a single center, which may limit the generalizability of our results to other populations. Moreover, due to its small sample size, our study is not able to establish a causal relationship between the clinical characteristics of wet AMD or the number of intravitreal injections and the aqueous level of neutralizing antibodies to aflibercept. Another significant limitation of our study is the absence of information on the patients’ concomitant cardiovascular and other non-cardiovascular conditions that may also contribute to changes in blood retina barrier permeability. Although these information would have helped elucidate the relationship between the morphology of AMD and the leakage of neutralizing antobodies into the ocular compartments, the primary purpose of this study was to assess the possibility to detect neutralizing antibodies in the aqueous samples. These results might help to make screening for optimal candidates for vector-based therapies more sensitive and to ensure that the treatment with anti-VEGF be more effective in these subjects.

In summary, repeated intravitreal aflibercept injections lead to the presence of nonspecific immunoglobulins in the aqueos humour, presumably due to entering the systemic circulation through the blood-retinal barrier. Further studies with larger sample sizes are recommended to assess the role of specific, neutralizing antibodies in the development of tachyphylaxis in patients with AMD.

## Data Availability

The datasets used and analysed during the current study available from the corresponding author on reasonable request.
